# Cell Size Checkpoint Control by the Retinoblastoma Tumor Suppressor Pathway

**DOI:** 10.1371/journal.pgen.0020167

**Published:** 2006-10-13

**Authors:** Su-Chiung Fang, Chris de los Reyes, James G Umen

**Affiliations:** Plant Biology Laboratory, The Salk Institute, La Jolla, California, United States of America; Washington University, United States of America

## Abstract

Size control is essential for all proliferating cells, and is thought to be regulated by checkpoints that couple cell size to cell cycle progression. The aberrant cell-size phenotypes caused by mutations in the retinoblastoma (RB) tumor suppressor pathway are consistent with a role in size checkpoint control, but indirect effects on size caused by altered cell cycle kinetics are difficult to rule out. The multiple fission cell cycle of the unicellular alga Chlamydomonas reinhardtii uncouples growth from division, allowing direct assessment of the relationship between size phenotypes and checkpoint function. Mutations in the C. reinhardtii RB homolog encoded by *MAT3* cause supernumerous cell divisions and small cells, suggesting a role for MAT3 in size control. We identified suppressors of an *mat3* null allele that had recessive mutations in *DP1* or dominant mutations in *E2F1,* loci encoding homologs of a heterodimeric transcription factor that is targeted by RB-related proteins. Significantly, we determined that the *dp1* and *e2f1* phenotypes were caused by defects in size checkpoint control and were not due to a lengthened cell cycle. Despite their cell division defects, *mat3, dp1,* and *e2f1* mutants showed almost no changes in periodic transcription of genes induced during S phase and mitosis, many of which are conserved targets of the RB pathway. Conversely, we found that regulation of cell size was unaffected when S phase and mitotic transcription were inhibited. Our data provide direct evidence that the RB pathway mediates cell size checkpoint control and suggest that such control is not directly coupled to the magnitude of periodic cell cycle transcription.

## Synopsis

All cell types have a characteristic size, but the means by which cell size is determined remain mysterious. In proliferating cells, control mechanisms termed checkpoints are thought to prevent cells from dividing until they have reached a minimum size, but the nature of size checkpoints has proved difficult to dissect. The unicellular alga Chlamydomonas reinhardtii divides via an unusual mechanism that uncouples growth from division, and thereby allows a direct assessment of how different genetic pathways contribute to size control. The retinoblastoma (RB) tumor suppressor pathway is a critical regulator of cell cycle control in plants and animals and is thought to act as a transcriptional switch for cell cycle genes, but it had not been directly implicated in cell size checkpoint function. The authors found that mutations in genes that encode key proteins of the RB pathway in *Chlamydomonas* affect cell size and cell cycle control by altering size checkpoint function. Unexpectedly, the predicted transcriptional targets of the RB pathway were not affected by the mutations, and blocking transcription did not alter cell size control. These data link the RB tumor suppressor pathway directly to size control and suggest the possibility that cell size and cell cycle control by the RB pathway may not be coupled to its transcriptional output.

## Introduction

Size control is a fundamental property of proliferating cells, but the mechanisms that maintain cell size are still poorly defined [1]. In budding and fission yeasts, size checkpoints are thought to govern either the G1/S or G2/M cell cycle transitions, preventing cells that have insufficient mass from continuing the cell cycle until they have grown to a minimum threshold size [2]. In animal cells, there is evidence for cell size checkpoint control [3–6], but the nature and existence of size checkpoints in animals are still debated [7]. In all eukaryotes, there appears to be a fundamental relationship between cell size and DNA content that is likely to be involved in governing cell size, but the nature of that relationship is still unknown [8].

Genome-wide screens have been carried out in budding yeast, yielding a large set of mutations that affect cell size [9,10], and similar screens were done with *Drosophila* tissue culture cells using RNA interference (RNAi) [11,12]. Mutants or RNAi knockdowns that alter growth rate or cell cycle progression show size phenotypes in such screens, but these phenotypes do not necessarily result from altered checkpoint control. Not surprisingly, cell size appears subject to input from multiple genetic pathways whose relative contributions to a size checkpoint mechanism are still unclear.

The retinoblastoma (RB) tumor suppressor pathway is conserved in most eukaryotic lineages, including animals, plants, and green algae, but has been lost from yeasts and other fungi. RB-related proteins interact with transcription factor heterodimers in the E2F and DP families and are thought to regulate cell cycle progression through the transcriptional activation or repression of genes required for S phase and mitosis [13–17]. Perturbation of RB pathway proteins has been shown to affect cell size and cell cycle control in animals and plants in a manner that is consistent with loss of size checkpoint control: Animals or plants that have lost RB or RB-related proteins usually have small cells [18–21], and overexpression of RB results in larger cells [22]. It is not clear whether these effects are due to altered size checkpoints or are caused by overall changes in cell cycle and/or growth rates that override checkpoint control. Moreover, a direct role for the RB pathway in size control is not precluded by the existence of its well-established role as an integrator of extracellular growth factor signals [15,23].

Chlamydomonas reinhardtii is a unicellular green alga that contains single copy genes encoding homologs of RB, E2F, and DP [24,25] and provides a simplified model for analysis of the RB pathway. One additional *Chlamydomonas* protein, E2FR1, has a single, highly diverged E2F-like DNA binding domain but no other conserved domains, and its function is unknown.

The C. reinhardtii multiple fission cell cycle is characterized by a long G1 period during which cells can grow in size by many fold. In early/mid G1, cells pass Commitment, a size checkpoint similar to Start in yeast or the restriction point in animal cells that gates passage through the remainder of the cell cycle. Cells that have passed Commitment will complete the cell cycle, even if subsequent growth is stopped by the withdrawal of light or nutrients, whereas pre-Commitment cells will return to a G0 resting state if growth is halted [26,27]. Different measurements in wild-type strains have determined that a minimum cell size must be attained in order to pass Commitment [25,26,28,29]. Post-Commitment cells do not immediately initiate S phase, but instead remain in G1 for an additional 5 to 10 h, where they can continue to grow and then finally undergo a rapid series of (n) alternating S phases and mitoses (S/M)_n_ to produce 2^n^ daughters. A critical aspect of size regulation occurs during S/M where mother cell size—which can vary over a wide range depending on growth conditions—controls the number of S/M cycles to produce a uniform size distribution of daughters [26,30]. Because S/M can occur in the absence of concurrent growth, daughter cell size can be used as a direct gauge of cell size checkpoint function [8].

The RB homologue in *C*. *reinhardtii* is encoded by the *MAT3* locus. *mat3* mutant cells pass Commitment at a prematurely small size, remain in G1 for several hours after Commitment (like wild-type cells), and then undergo supernumerous divisions to produce daughters that are 25% to 35% the size of wild-type daughters [25,31]. Thus, similar to its role in animals and plants, MAT3/RB acts as a negative regulator of cell cycle progression. While it was postulated to be involved in cell size checkpoint control, it was not clear whether MAT3 acted through DP and E2F proteins and, if so, whether E2F and DP controlled rate limiting steps in cell size regulation.

Here we identified bypass suppressors of an *mat3* null allele that have mutations in the *Chlamydomonas DP1* and *E2F1* genes. We found that *dp1* null mutants had a large-cell phenotype caused by an increased Commitment threshold size and a failure to initiate enough rounds of division during S/M, opposite to the size checkpoint phenotypes of *mat3* mutants. Two classes of dominant *e2f1* suppressors were found that displayed different degrees of severity of cell cycle and cell size phenotypes. Importantly, the suppression of *mat3* by *dp1* and *e2f1* mutations was not due to a lengthened cell cycle but rather to a defect in size checkpoint function. Surprisingly, however, we found no significant transcriptional defects for periodically expressed cell cycle genes in *dp1, e2f1,* or *mat3* mutant strains. Moreover, we found that inhibition of periodic cell cycle transcription had no effect on the number of cell divisions carried out by either wild-type or *dp1* mother cells and no influence on daughter cell size. These data suggest that the RB pathway controls size-dependent cell division in a manner that is not quantitatively linked to transcriptional output during cell division.

## Results

### Isolation and Characterization of *mat3* Bypass Suppressors

**Figure 1:**
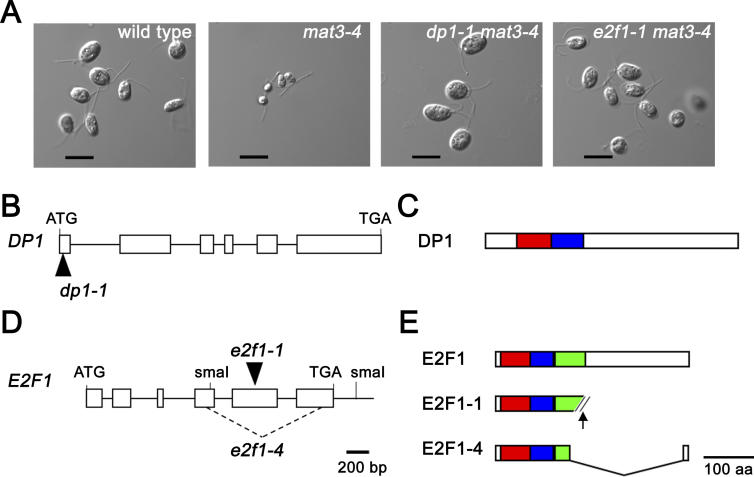
Suppressors of *mat3–4* (A) Nomarski images of daughter cells from indicated strains. Scale bar = 10 μm. (B, D) Schematic of *DP1* and *E2F1* loci, with exons and introns denoted by rectangles and solid lines, respectively. Translation start (ATG) and stop (TGA) codons, and SmaI cut sites are shown. Plasmid insertion sites for *dp1–1* and *e2f1–1* alleles are indicated by dark triangles. The deleted region of *e2f1–4* genomic DNA is indicated by a dashed line. (C, E) Schematics of DP1 and E2F1 proteins showing DNA binding domains (red), dimerization domains (blue), and Marked box (green). The arrows indicate the position where the *e2f1–1* insertions would interrupt the peptide sequence. For E2F1–4, part of the predicted E2F1 protein is missing due to an in-frame deletion as indicated.

To investigate the downstream targets of MAT3 that are required for regulation of cell division, we carried out an insertional mutagenesis screen for bypass suppressors of a null allele, *mat3–4* [25,32]. Approximately 20,000 insertion lines were screened, from which 19 suppressors were isolated, including 12 *DP1* alleles and three *E2F1* alleles, all of which showed strong suppression: The *dp1* mutants caused a large-cell phenotype, while the *e2f1* mutants restored cells to approximately wild-type size (Figure 1A and Table 1). Four partial suppressors were isolated that had a more modest effect on *mat3–4* cell size (unpublished data), and these suppressors will be described elsewhere along with details of the screen (S.-C. Fang and J. G. Umen, unpublished data). No other strong suppressors were found, but there are likely to be additional partial suppressors since none of those found were allelic. Mutations in the *E2FR1* locus were not recovered in this screen.

**Table 1:**
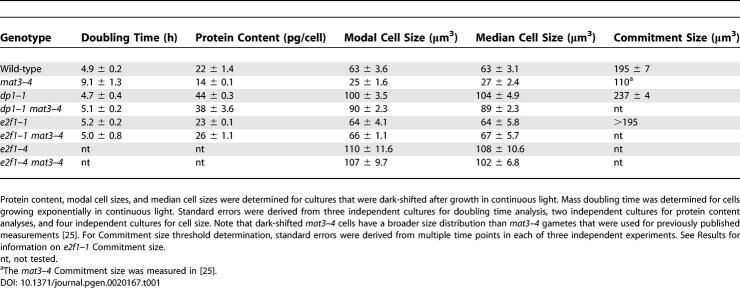
Growth Rate, Protein Content, Cell Size, and Commitment Size Threshold Data for Indicated Strains

Some of the *DP1* alleles contained deletions that completely removed the locus and some surrounding DNA, while two alleles contained insertions without any other detectable alterations (Figures 1B and S1 and unpublished data). Reference allele *dp1–1* contains a 4.7-kb plasmid insertion in the first exon (Figures 1B and S1) that is predicted to block production of a functional protein (Figure 1C). *dp1–1* behaved indistinguishably from a deletion allele, *dp1–3* (Figure S3 and unpublished data), and was used for most of the experiments.

The three *e2f1* suppressor mutants isolated in this screen all contained insertions in the 3′ end of the *E2F1* gene (Figures 1D and S1) that would be predicted to allow production of a C-terminally truncated protein that retains DNA binding, dimerization, and Marked Box domains (Figure 1E). These three *E2F1* alleles—*e2f1–1, –2,* and *–3*—behaved identically, and reference allele *e2f1–1* (Figure 1D) was used for most of the experiments. A fourth suppressor allele, *e2f1–4*, was isolated fortuitously from a population of transformed *mat3–4* cells and contains an internal deletion that removes exon 5 and fuses parts of exons 4 and 6 in-frame (Figure 1D and see Materials and Methods). The phenotype of *e2f1–4* is described in more detail below. Based on their repeated isolation, the screen appears to be saturated for the *DP1* and *E2F1* loci. Possible reasons for the absence of obvious *e2f1* null alleles are presented in the Discussion.

**Figure 2:**
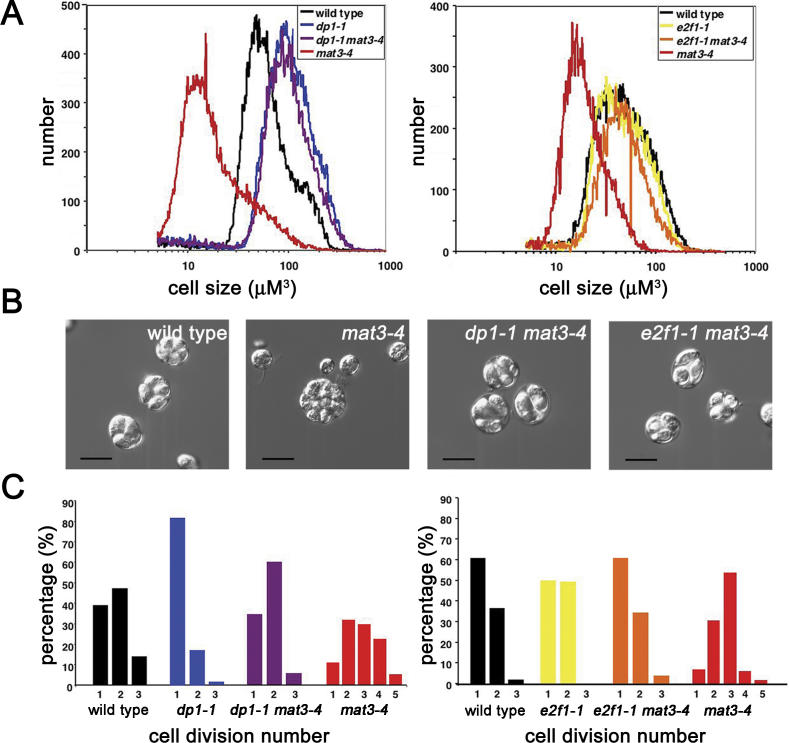
*e2f1* and *dp1* Mutations Suppress *mat3–4* Cell Division Phenotypes (A) Cell size distributions of dark-shifted cultures from indicated strains. (B) Nomarski images of postmitotic clusters from indicated strains. Scale bar = 10 μm. (C) Distribution of cell division numbers from dark-shifted post-Commitment cells of indicated genotype.

In principle, any mutation that slows cell cycle progression might increase cell size and suppress *mat3–4*. We therefore carried out dark-shift assays to determine whether suppression of *mat3–4* by *dp1–1* and *e2f1–1* was due to a slowed cell cycle or due to a fundamental change in the size checkpoint function that couples mother cell size to the proper number of division cycles. Unsynchronized cultures of wild-type, single, and double mutants were grown in continuous light to generate a random distribution of cells and then shifted into the dark for 16 to 18 h. Upon shifting to the dark, growth ceases, and any cell that is past Commitment will enter S/M phase (typically within 5 to 10 h for wild-type) and divide to produce 2^n^ daughter cells. Given sufficient time, even “slow” cell cycle mutants will eventually produce daughter cells of the appropriate size [8,33]. However, the suppressed strains always produced daughters whose sizes were larger than wild-type for *dp1–1 mat3–4* or similar to wild-type for *e2f1–1 mat3–4*, regardless of how long they were dark shifted (Figure 2A, Table 1, and unpublished data). These results indicated that both *dp1–1* and *e2f1–1* suppressed *mat3–4* by altering the size checkpoint function that controls how many S/M cycles are initiated rather than simply slowing cell cycle progression. The cell cycle kinetics for *dp1–1* and *e2f1–1* are described further below.

We also observed the cell cycle behaviors of *dp1–1* and *e2f1–1* single mutants that were generated by outcrossing each suppressed double mutant to a wild-type strain. We carried out dark-shifting experiments for each single mutant and found that their daughter cell sizes were almost identical to the corresponding double mutant strains with *mat3–4* (Figure 2A and Table 1). This finding suggested that the *dp1–1* and *e2f1–1* strains were largely insensitive to the presence or absence of MAT3/RB and that the deregulated cell division phenotypes of *mat3–4* null strains were dependent on DP1 and E2F1. Based on their epistatic relationships with *mat3–4*, the different daughter cell sizes of *dp1–1* and *e2f1–1* mutants seemed somewhat paradoxical, but this difference is likely to be caused by residual cell cycle activation that can occur in *e2f1–1* strains. This idea is elaborated further in the Discussion.

We investigated the cell division behavior for each mutant strain more directly by plating unsynchronized light-grown cells on agar, shifting the plates to the dark, and then assaying cell division for individual cells after dark incubation [25]. While post-Commitment *mat3–4* cells almost always divided at least two and sometimes up to five times under these conditions, the suppressed strains had division numbers that were closer to wild-type, with most committed cells dividing either one or two times (Figure 2B and 2C). While the *dp1–1* cell division numbers appeared similar to wild-type in these experiments, they were abnormally low given the larger size of *dp1–1* or *dp1–1 mat3–4* mother cells (Figure 2C and unpublished data). Taken together, our data show that *dp1* and *e2f1* mutations block the ability of *mat3* mutant cells to initiate extra rounds of S phase.

Commitment is a cell size checkpoint that is also controlled by the RB pathway in *Chlamydomonas*. *mat3* mutants were previously shown to pass Commitment at a smaller size than wild-type cells [25], and we therefore asked whether *dp1* or *e2f1* mutants also had altered Commitment cell size thresholds. Commitment was measured in synchronous cultures so that G1 populations with well-defined size ranges could be obtained. Synchrony for *dp1* and *e2f1* strains was much more difficult to achieve than for wild-type, but we were able to synchronize the mutants using a modified light-dark regimen that also worked for wild-type cells (see Materials and Methods).

**Figure 3:**
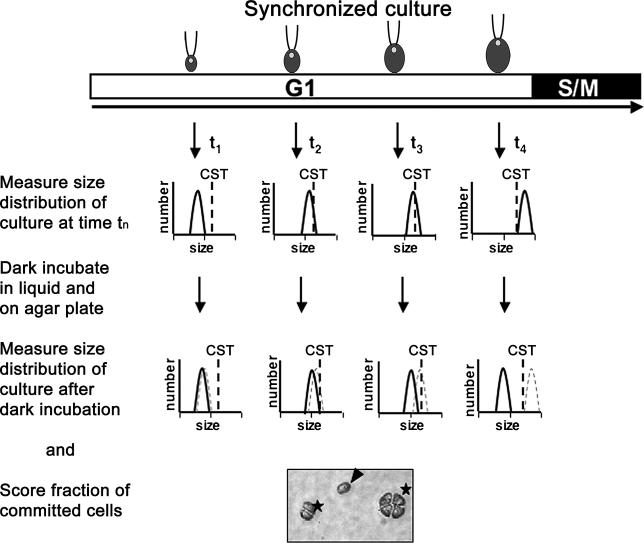
Schematic Illustration of Commitment Size Threshold Assay The light/dark phases of synchronized cultures are indicated by the white and black bar on top. Aliquots of G1 cells are removed at different times (t_1_, t_2_, t_3_, t_4_), for size measurements (schematized in top row), and for dark-incubation in liquid and on plates. The dashed vertical line labeled CST represents a potential *C*ommitment *S*ize *T*hreshold. After dark-incubation for 18 to 24 h in liquid, the daughter cell size distribution is measured (schematized in lower set of size distribution curves). The light-colored dashed curve represents the size distribution prior to the dark shift, and the dark solid line represents the cell size distribution after dark shifting and cell division. The aliquot of cells that was incubated on plates in the dark was scored microscopically to determine the fraction of committed cells in the population. The arrowhead in the picture inset indicates a cell that was not committed, and the stars indicate cells that were committed and divided either once or twice to produce two and four daughters, respectively. The CST is empirically determined by finding a size threshold value that best explains the behavior of the cells with respect to the fraction that were committed in each sample. The reliability of this value is reflected in the standard error (see Table 1).

**Figure 4:**
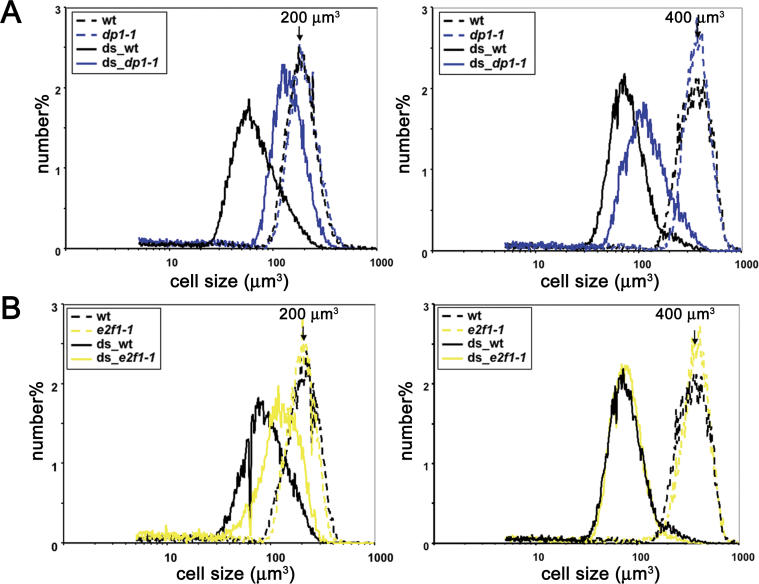
Commitment Experiments with Synchronized Wild-Type, *dp1–1,* and *e2f1–1* (A) Size distributions of synchronized cells growing in the light (dashed lines) and after dark-shifting (ds) (solid lines) for wild-type (black lines) and *dp1–1* (blue lines) cultures at two different times during G1 (left and right panels). In the left panel, the preshifted cells have a modal size of approximately 200 μm^3^, and in the right panel, they have a modal size of approximately 400 μm^3^. (B) Size distributions of synchronized cells growing in the light (dashed lines) and after dark-shifting (ds) (solid lines) for wild-type (black lines) and *e2f1–1* (yellow lines) cultures at two different times during G1 (left and right panels). In the left panel, the preshifted cells have a modal size of approximately 200 μm^3^, and on the right panel, they have a modal size of approximately 400 μm^3^.

The Commitment size threshold in the synchronous cultures was measured by removing aliquots of cells at various times during G1 and determining their cell size distributions before and after dark-shifting, and by plating aliquots of cells on agar in the dark in order to more accurately determine the fraction of committed cells (Figure 3). The data on cell size distribution and fraction of committed cells were then combined to determine the threshold size at which cells divide. For wild-type cultures, as cells approached approximately 195 μm^3^, they passed Commitment and were capable of dividing after a dark-shift. In other words, the fraction of cells that was larger than approximately 195 μm^3^ accurately predicted the fraction of cells in the population that were committed (Figure 4A and Table 1). The wild-type Commitment size we measured was slightly larger than that previously reported of approximately 178 μm^3^ [25], but this difference may be due to the method of cell size measurement employed (Coulter Counter versus microscopic) and is within the margins of error for such measurements. Compared to wild-type, *dp1–1* cells were found to reach a larger size of approximately 237 μm^3^ before they could pass Commitment (Figure 4A and Table 1). Thus, *dp1–1* cells showed defects in both passing Commitment and in regulating daughter cell size.

*e2f1–1* mutants displayed an interesting behavior that we uncovered in synchronous cultures but that was not readily apparent in dark-shift assays using unsynchronized cultures. As *e2f1–1* cells reached wild-type Commitment size, they divided much less readily, and if they divided, produced large daughters compared with wild-type cells (Figure 4B, left). However, as the cells continued to grow and reached sizes well above wild-type Commitment size, they could produce daughters similar in size to wild-type daughters (Figure 4B, right). Thus, *e2f1–1* mutants had an increased Commitment size, and cells that were just past Commitment also appeared to have defects in daughter cell-size control. However, the daughter cell-size defect disappeared as cells grew larger and the entire population became committed. While *e2f1–1* mutants clearly had a larger Commitment size than wild-type, measuring their Commitment size proved difficult. Unlike wild-type and *dp1–1* cultures, synchronized *e2f1–1* cultures behaved somewhat unpredictably, sometimes passing Commitment just above the threshold size of wild-type cells and sometimes considerably later. This instability suggested that *e2f1–1* cells had a more severe defect early in the cell cycle but that this defect could be partially overcome with continued growth. However, it is important to note that the supernumerous cell divisions of *mat3–4* were effectively suppressed by *e2f1–1* under all circumstances, meaning that even the apparently normal S/M cycles seen in *e2f1–1* strains concealed an underlying defect.

### Complementation and Dominance Testing

**Figure 5:**
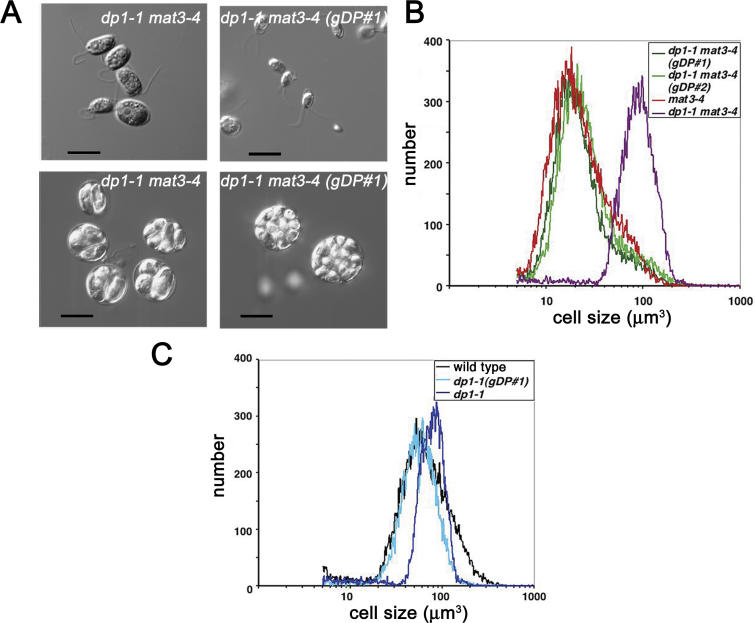
Complementation of *dp1–1* (A) Nomarski images of *dp1–1 mat3–4* double mutants with and without a complementing genomic construct containing the wild-type *DP1* gene (gDP#1). Top panels show dark-shifted cells, and bottom panels show postmitotic clusters. Scale bar = 10 μm. (B) Size distributions of dark-shifted cells from *mat3–4*, *dp1–1 mat3–4*, and two independently generated *dp1–1 mat3–4* transformants that are complemented with gDP1 (gDP#1 and gDP#2). (C) Size distributions of dark-shifted wild-type, *dp1–1,* and complemented *dp1–1* (gDP#1) strains.

We attempted to complement the *dp1–1* and *e2f1–1* strains in order to confirm that the mutant phenotypes we observed were due to defects in each of these loci. To complement *dp1–1,* we started with a *dp1–1 mat3–4* strain and transformed in a genomic construct containing the wild-type *DP1* gene (gDP1). Several gDP1 transformants had an *mat3–4*–like cell size distribution, indicating that the *dp1–1* suppressor phenotype had been complemented (Figure 5A and 5B). The complemented strains were then crossed to a wild-type strain to confirm linkage of gDP1 to the complemented phenotype and to segregate it away from *mat3–4*. All the *mat3–4 dp1–1* progeny from the cross that also had gDP1 were tiny, whereas all those that did not receive gDP1 were large (unpublished data). Moreover, all the *dp1–1* single mutant progeny that received gDP1 were wild-type in size, whereas those that did not receive gDP1 were large (Figure 5C). Finally, there were no size differences among wild-type progeny regardless of whether they received the gDP1 construct (unpublished data). These data confirm that loss of DP1 is responsible for suppressing *mat3–4* and for causing the large-cell phenotype that we observed in *dp1* mutant strains. The transformation experiments also showed that increased dosage of *DP1* in a wild-type strain has no obvious phenotype.

**Figure 6:**
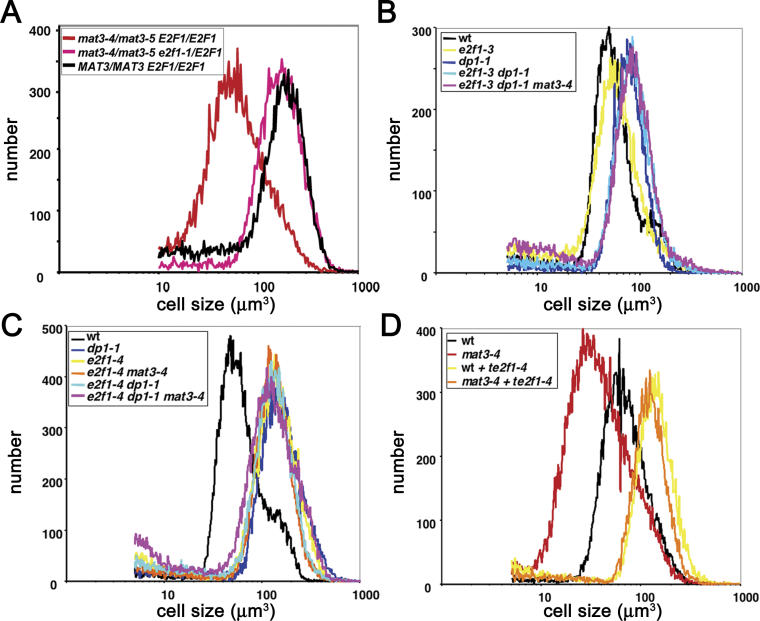
Dominance Testing and Genetic Interactions of *e2f1* Mutants (A) Size distributions of dark-shifted vegetative diploids of indicated genotype. (B) Size distributions of dark-shifted wild-type, *e2f1–3*, *dp1–1*, *e2f1–3 dp1–1,* and *e2f1–3 dp1–1 mat3–4* strains. (C) Size distributions of dark-shifted wild-type, *dp1–1*, *e2f1–4*, *e2f1–4 mat3–4, e2f1–4 dp1–1*, and *e2f1–4 dp1–1 mat3–4* strains. (D) Size distributions of dark-shifted wild-type and *mat3–4* with and without a transgenic *e2f1–4* allele (*te2f1–4*).

Unlike the case for *dp1–1,* we were unable to complement *e2f1–1,* suggesting that it might encode a dominant allele whose gene product cannot interact with MAT3. This explanation is conceivable since the insertion that interrupts the sequence of *e2f1–1* occurs downstream of the region encoding its DNA binding, dimerization, and Marked Box domains and upstream of the region that encodes an RB-interacting domain in other E2F proteins (Figure 1D and 1E). Indeed, we found that an *mat3/mat3 e2f1–1/E2F1* diploid strain was similar in size to a wild-type diploid strain (approximately 2-fold larger than a wild-type haploid) and was not the size of an *mat3/mat3 E2F1/E2F1* diploid strain as would be expected if *e2f1–1* were recessive to *E2F1* (Figure 6A). The fact that the three *e2f1* mutants we isolated had less severe cell size phenotypes than the *dp1* mutants suggests that the mutant E2F1 proteins retain some residual ability to activate the cell cycle. This residual activity was dependent on the presence of DP1 since *e2f1–3 dp1–1* and *e2f1–1 dp1–2* mutants were identical in size to *dp1–1* single mutants (Figure 6B and unpublished data). The lack of synergistic or additive genetic interactions between *e2f1–1* and *dp1–1* suggests that they act together or on a common set of targets to suppress *mat3–4*.

In order to better establish the dominant nature of truncated *E2F1* alleles, we took advantage of a fortuitously isolated allele, *e2f1–4,* that arose in an *mat3–4* strain during a transformation experiment (see Materials and Methods). Similar to the other *E2F1* alleles, the protein encoded by *e2f1–4* is predicted to lack most its C-terminal sequence; but unlike the other three alleles, the predicted *e2f1–4*-encoded protein is also missing part of its Marked Box, a conserved domain in E2F proteins that is adjacent to the dimerization domain, and which mediates interaction with both RB-related proteins [34,35] and with co-factors [36–40] (Figure 1D and 1E). *e2f1–4* has a more severe cell size defect than *e2f1–1, –2,* and *–3,* producing large daughters that are similar in size to *dp1* daughter cells (Figure 6C). However, like the other three *E2F1* alleles, *e2f1–4* does not show any genetic interactions with *dp1–1* and is completely epistatic to *mat3–4* (Figure 6C).

We constructed a genomic library from the *e2f1–4* strain and subcloned an approximately 4-kb fragment that contains the *e2f1–4* allele. The *e2f1–4*–containing construct was then reintroduced into an *mat3–4* strain by transformation. Whereas in a control transformation with an empty vector, no suppressed transformants were identified, several of the *mat3–4* transformants that received an *e2f1–4* construct had a suppressed, large-cell phenotype, similar to the original *e2f1–4* strain. Two such suppressed transformants were crossed to a wild-type strain, and the progeny were scored for their size phenotypes. We found that all the *mat3–4* progeny that received the *e2f1–4* transgene construct showed a large-cell phenotype, whereas the *mat3–4* progeny that had no *e2f1–4* transgene were small (Figure 6D). Interestingly, progeny that were wild-type for *MAT3* and had an *e2f1–4* transgene also had a large-cell phenotype (Figure 6D). These transformation experiments demonstrate directly that truncated *e2f1* alleles can dominantly suppress *mat3–4* and that such alleles can also interfere with size control in a wild-type strain, generating a large-cell phenotype.

### Cell Cycle Transcription in *dp1*, *e2f1,* and *mat3* Mutants

**Figure 7:**
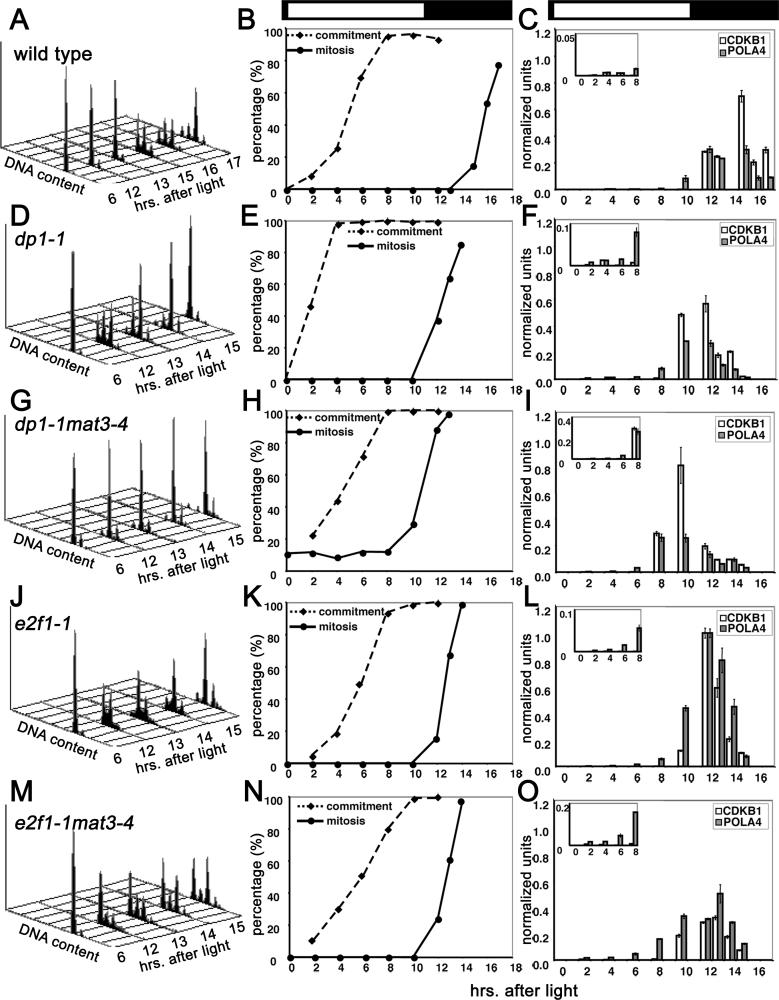
Cell Cycle Kinetics and Gene Expression Patterns in Synchronized Wild-Type and Mutant Strains (A, D, G, J, M) FACS profile showing the DNA content of different genotypes during cell cycle progression. Note that postmitotic clusters often remain together after division and account for the persistence of multiple peaks in some strains. (B, E, H, K, N) Graphs showing passage through Commitment (black diamonds with dashed lines) and mitotic index (black circles with solid lines) of synchronous cultures. Wild-type cultures entered S/M phase at approximately 13 h. Cultures of *dp1–1*, *dp1–1 mat3–4*, *e2f1–1*, and *e2f1–1 mat3–4* passed Commitment and entered the S/M phase earlier. (C, F, I, L, O) Expression of S/M phase markers *CDKB1* and *POLA4* by quantitative RT-PCR. Insets show rescaled graphs to visualize expression at early time points. The light/dark phases are indicated by white or dark bars above the graphs. Expression was normalized to the cytoplasmic 18S rRNA signal in each sample. Data are from technical triplicates and presented as mean normalized units ± SEM.

**Figure 8:**
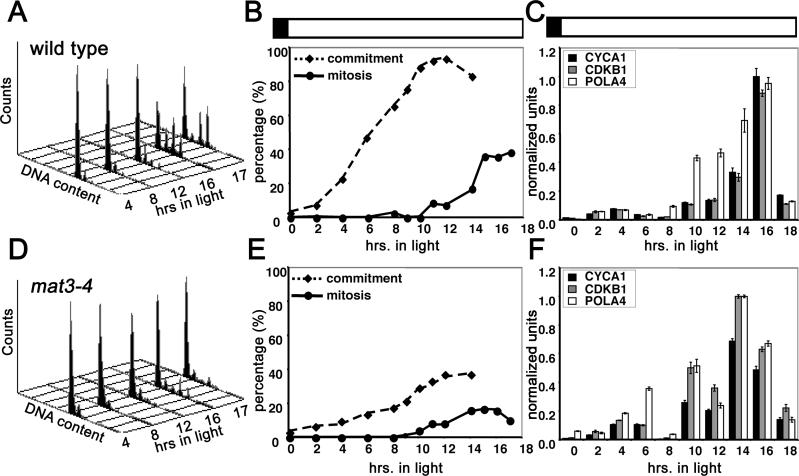
Cell Cycle Kinetics and Gene Expression in Partially Synchronized Cultures of Wild-Type and *mat3–4* The light/dark phase is indicated by white or dark bars above the graphs. (A, D) FACS profile showing the DNA content of wild-type and *mat3–4* strains that were dark shifted and returned to continuous light. (B, E) Graphs show Commitment (black diamonds with dashed lines) and mitotic index (black circles with solid lines) of the cultures. Both wild-type and *mat3–4* cultures entered S/M phase at approximately 8 to 10 h after return to the light and reached a mitotic peak at approximately 14 to 16 h. (C, F) Expression of S/M phase markers *CYCA1*, *CDKB1,* and *POLA4* by quantitative RT-PCR as described in Figure 7.

In *C. reinhardtii,* mRNA levels of cell cycle regulators as well as genes involved in DNA synthesis are transcribed periodically, with peak expression occurring during S/M [24]. We therefore tested whether induction of these periodically expressed genes was altered in mutant strains (Figures 7 and 8). Based on work in other organisms, we predicted decreased transcript levels in *dp1* mutants, increased transcript levels in *mat3* mutants, and decreased or absent transcriptional periodicity in all the mutants.

During synchronous growth, the mutant strains remained in G1 for a shorter time than wild-type, entering S/M 2 to 4 h earlier (Figure 7A, 7B, 7D, 7E, 7G, 7H, 7J, 7K, 7M, and 7N), but like wild-type, all mutants remained in G1 for several hours after passing Commitment and before initiating S/M (Figure 7B, 7E, 7H, 7K, and 7N). Importantly, *dp1* and *e2f1* mutants completed the S/M phase within 3 to 4 h, similar to wild-type cells (Figure 7A, 7B, 7D, 7E, 7G, 7H, 7J, 7K, 7M, and 7N), indicating that they were not severely impaired in progressing through S phase and mitosis.

The availability of synchronous cultures of *dp1* and *e2f1* mutants allowed us to measure kinetics of periodically expressed cell cycle genes. We chose genes that matched several criteria, including strong periodic expression during S/M [24], conservation as E2F targets in plants and/or animals [15,41–43], and the presence of one or more potential E2F consensus sites near the predicted transcription start site [44]. These genes included ribonucleotide reductase *(RIR1),* PCNA *(PCN1),* DNA primase *(POLA4),* cyclin A *(CYCA1),* cyclin B *(CYCB1),* CDKB *(CDKB1), CDC6, MCM2,* and histone H4 *(HFO)* (a multicopy gene family). With the exception of histone H4 (no site found) and *CDKB1* (approximately 800 base pairs upstream), each of these genes has at least one consensus E2F binding site within 400 base pairs of its predicted transcriptional start site (unpublished data) and could be a target of the pathway.

In synchronous wild-type cultures, these cell cycle genes were repressed during G1 and induced by as much as 100-fold during S/M (Figures 7C and S4). Unexpectedly, a similar pattern of repression and induction was observed in *dp1* and *e2f1* strains alone or in combination with *mat3–4*, with peak expression levels comparable to what was observed for wild-type (Figures 7F, 7I, 7L, 7O, and S4). Thus, despite their defects in initiating the correct number of S phases, *dp1* mutants showed no defects in inducing or repressing the transcriptional program that accompanies S/M. Moreover, *dp1* and *e2f1* mutants did not display any delay in initiating S/M phase after passing Commitment (Figure 7D, 7E, 7G, 7H, 7J, 7K, 7M, and 7N) as might be expected if rate-limiting transcripts were slow to accumulate. On the contrary, the *dp1–1* and *e2f1–1* cultures entered S/M slightly earlier than the wild-type culture. In summary, the *dp1* and *e2f1* mutant strains showed slightly altered G1 cell cycle kinetics compared to wild-type but initiated and completed S/M at a similar rate to wild-type, with an apparently normal transcriptional program for all genes that were tested.

*mat3–4* single mutants could not be synchronized by the methods used for the other strains, so in parallel with a wild-type strain, we induced partial synchrony by dark-shifting a continuous-light culture, followed by a return to continuous light [25]. Under these conditions, entry into S/M phase was less synchronous than it was for the diurnally cycled cultures, with the peak mitotic index for wild-type at approximately 38% and for *mat3–4* at approximately 18% (Figure 8A, 8B, 8D, and 8E). For both wild-type and *mat3–4* cells, the G1 repression of periodic cell cycle transcripts was less pronounced than in highly synchronous cultures (Figure 8C, 8F, and S5), suggesting that tight transcriptional control during G1 is not critical for regulating cell cycle progression. While messages for some cell cycle genes in *mat3–4* strains were slightly elevated compared to wild-type (Figures 8C, 8F, and S5), overall the effect of the mutation on cell cycle gene transcription was modest and unlikely to account for the severe loss of replication control displayed by *mat3–4* mutants*.*

### Inhibition of Periodic Cell Cycle Transcription in Wild-Type and *dp1* Mutants

**Figure 9:**
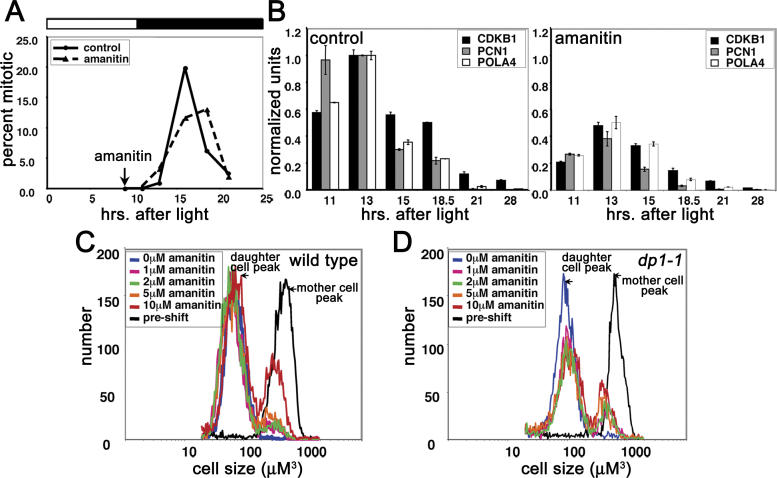
Cell Division in the Presence of RNA Polymerase II Inhibitor α-Amanitin (A) α-Amanitin 10 μM was added to 12:12 light/dark synchronized wild-type cells at 9 h in the light period (vertical arrow), and the drug-treated or control cultures were dark-shifted at 10 h. The cells stayed in the dark for the remainder of the experiment. The mitotic index was measured for each culture as indicated. (B) Quantitative RT-PCR of S/M phase markers *CDKB1*, *PCN1,* and *POLA4* from control and α-amanitin–treated cultures at different times. (C, D) Dose response of synchronized wild-type (C) or *dp1–1* (D) cells treated with different concentrations of α-amantin prior to cell division. Mother cell size distribution is shown by the black curve (pretreatment), and postmitotic daughter cell sizes produced in different concentrations of α-amantin by the different color curves.

Our results showing little difference in periodic cell cycle transcription with RB pathway mutants suggested that their underlying defects might not be linked to S/M transcription levels. Therefore, in order to test more broadly whether any periodically transcribed genes were important for determining the number of rounds of replication during S/M, we used the RNA polymerase II inhibitor α-amanitin (Figure 9). We treated a synchronized culture of wild-type cells in mid-G1 (post-Commitment) with 10 μM α-amanitin, a dose that was sufficient to completely block cell proliferation within one or two generations (unpublished data). At this dose, a fraction (10% to 20%) of the drug-treated cells failed to enter S/M (Figure 9C), suggesting that initiation of the S/M program depends on accumulation of a limiting transcript(s). The progression of the remaining cells through S/M was delayed slightly by α-amanitin (Figure 9A), but, importantly, despite decreased cell cycle transcription (Figure 9B), the daughter cell-size profile of the α-amanitin–treated culture was essentially identical to that of the control cells (Figure 9C, μM amanitin). This experiment suggested that the program which determines how many rounds of S phase are initiated within each cell cycle and which is controlled by the MAT3/RB pathway is insensitive to the message levels of cell cycle transcripts.

We also compared the dose response of wild-type and *dp1–1* cells to α-amanitin, reasoning that if *dp1–1* mutants were limiting for S phase transcription, then their daughter cell-sizes would be particularly sensitive to the drug. We administered different doses of α-amanitin to synchronous wild-type and *dp1–1* cultures in mid-G1 and then observed the size distribution of daughter cells at the end of the dark period. Like wild-type, the decision to enter S/M was sensitive to α-amanitin in *dp1–1* strains, but once they entered the replication phase of the cell cycle, the α-amanitin–treated *dp1–1* cells completed the same number of divisions as untreated cells, and produced daughters whose size profiles were essentially the same as untreated *dp1–1* (Figure 9C and 9D). Therefore, S/M transcription does not appear to be rate limiting for S phase control, even in *dp1* mutants.

The MAT3/RB pathway also controls passage through Commitment, but because administration of α-amanitin blocks some subsequent cell division, we did not attempt to assess its effects earlier in the cell cycle. *mat3–4* single mutant strains were found to respond to low doses of cytotoxic agents (e.g., actinomycin, zeocin, methyl methanesulfonate) by producing slightly larger daughters (unpublished data), but these effects were not dosage sensitive and instead may reflect a stress response. This response was not seen in *dp1* or *e2f1* strains but dissuaded us from further drug testing with *mat3–4* mutants.

## Discussion

### The Genetic Architecture of the RB Pathway in *Chlamydomonas*

The RB pathway is found in several eukaryotic groups, including animals and plants, whose last common ancestor was unicellular [45]. Previous findings indicating that MAT3/RB is a negative cell cycle regulator in *Chlamydomonas* were consistent with the idea that the cell cycle regulatory function of the RB pathway was already established prior to the formation of the current major eukaryotic taxa [25]. Here we have shown that the key targets of MAT3/RB-mediated cell cycle activation are homologs of E2F and DP. While this finding may seem obvious in retrospect, it provides the strongest evidence to date for the common origin and functional conservation of the RB pathway in an ancestral eukaryotic lineage. It also establishes *Chlamydomonas* as a simple, unicellular model for the RB pathway.

The RB pathway is clearly essential for development in plants and animals, but one critical question that our work addresses is whether E2F-DP activity has an essential cell-autonomous role in driving the cell cycle. In *Drosophila,* some larval development and cell cycle progression can occur in DP mutants [46,47], but it is not clear to what extent maternal stores of protein might allow mutant larval cells to cycle. In mammalian cells, the activity of E2F1, E2F2, and E2F3 was proposed to be required for proliferation [48], but this requirement may be based on an imbalance caused by loss of positive but not negative E2Fs [49]. In *C. elegans,* the RB pathway appears to be primarily required for developmental regulation and has a largely redundant role in regulating the cell cycle [50,51]. Based on genetic interactions in other species and on our own genetic and in vitro data (K. Bisova and J. G. Umen, unpublished data), DP1 and E2F1 function as heterodimers, and loss of DP1 is expected to eliminate E2F1 activity. Although it is difficult to completely rule out redundancy, our data are most simply interpreted as E2F1-DP1 heterodimers having a nonessential role in cell cycle progression, but a critical role in maintaining size homeostasis.

However, it is formally possible that other E2F-like genes exist in *Chlamydomonas* that compensate either for loss of *DP1* or dominant mutations in *E2F1*, but several observations suggest that this is not the case. First, in the current and all previous *Chlamydomonas* genome assemblies, extensive bioinformatic searching has never identified any E2F or DP family members other than those already described [24]. Second, no other copies of *E2F1* or *DP1* were detected by low stringency Southern blotting (Figure S2). Third, a comprehensive search of the closely related Volvox carteri genome revealed single orthologs of *Chlamydomonas* E2F1, DP1, and E2FR1, but no additional E2F/DP family members (unpublished data). While it has some resemblance to E2F/DP like proteins, E2FR1 appears to be a poor candidate for an E2F1 or DP1 substitute—its DNA binding domain is diverged, and it contains no obvious dimerization domain. Nonetheless, it may be able to substitute in some way for the loss of DP1 or dominant mutation in E2F1. If it did so (either as a binding partner or in some other way), then we might have expected to see added phenotypic severity when *dp1* and *e2f1* mutations were combined, but that was never the case. We are currently attempting to isolate null alleles of *E2F1* and RNAi lines for *E2FR1* so that this question can be resolved more conclusively.

Two unusual aspects of the suppressor screen were the high rate of isolation of both *DP1* and *E2F1* alleles (12 of 20,000 and three of 20,000, respectively), and the absence of *e2f1* null mutations. If insertions were random, then they would occur on average every 5 kb among 20,000 mutants (for an approximately 100-Mb genome) and should have generated one allele each for *DP1* and *E2F1*. Thus, both the *DP1* and *E2F1* loci appear to be hotspots for insertions/deletions. Whether this insertion bias is also present in a wild-type strain background is not known. It is notable that all the *E2F1* insertions we identified were in a 1.4-kb SmaI fragment (Figures 1D and S1) and that *e2f1–4* resulted from a spontaneous deletion in the same region (Figure 1D). While it is possible that *E2F1* is essential, our lack of null alleles in the screen cannot be taken as evidence that this is the case since the locus was highly susceptible to mutations in the 3′ region that caused a dominant suppressor phenotype.

The structure of the *E2F1* alleles that we isolated is consistent with them acting as dominant negatives. As has been observed elsewhere, C-terminally truncated alleles of E2F can interfere with E2F function in mammalian cells [52]. Interestingly, a similar allele from *Drosophila, de2f^i2^,* has transcriptional defects [53] and can suppress the loss of the fly RB homolog, *RBF1* [46], but is recessive [53]. The complete dominance we observed for *Chlamydomonas* E2F1 suppressor alleles could be due to enhanced stability of the mutant E2F1 proteins or mRNA compared to wild-type, but this remains to be determined.

While the C-terminal region of *Chlamydomonas* E2F1 lacks an obvious RB binding domain that is found on other E2Fs [49,54,55], we propose that it encodes an alternative motif that interacts with MAT3/RB. We also propose the existence of a residual cell cycle activation function in heterodimers formed between *e2f1–1, −2, −3* encoded proteins and DP1 that generates a partially functioning size control mechanism. The mutant heterodimers may, for example, allow the slow accumulation of a rate-limiting cell cycle activity so that in large mother cells this activity is sufficient to support a normal number of S/M cycles. At the same time, this class of *E2F1* alleles must also limit the excess rounds of replication and division that occur in *mat3* mutants, perhaps by restricting the overaccumulation of the same rate-limiting activity that occurs when MAT3/RB is missing.

### The RB Pathway and Cell Size Control

Our genetic results demonstrate a role for DP1-E2F1 and MAT3/RB as positive and negative regulators of the cell cycle, respectively, that affect cell size in opposite ways. While this observation might be construed as a trivial outcome of shortened or lengthened cell cycles, this is not the case. In *Chlamydomonas,* the effects on cell size checkpoints can be uncoupled from cell cycle kinetics, and the results we obtained clearly implicate the RB pathway in cell size checkpoint control. As previously observed, *mat3* cells pass Commitment prematurely and then divide too many times during S/M [25]. Here, we showed that *dp1* and *e2f1* mutants have the opposite phenotype of *mat3,* passing Commitment at a larger size and exiting S/M prematurely before an adequate number of cell divisions have been completed. Moreover, *dp1* mutants did not appear to remain in S/M for an unduly long period of time, and they were not slow to transition from G1 into S/M; on the contrary, *dp1* mutants spent less time in G1 than wild-type cells. Nonetheless, *dp1* cells maintained a large size because they failed to initiate enough rounds of S phase. Therefore, the effects we saw on cell size in RB pathway mutants were due to aberrant cell size checkpoint control and were not caused by a slow cell cycle.

The fact that cells missing key components of the RB pathway can still maintain size equilibrium, albeit at abnormal sizes, indicates that there are alternative mechanisms that can contribute to cell size homeostasis. Therefore, the rate-limiting factors that are required for cell cycle progression can be produced in cells that are mutant for *MAT3, E2F1,* or *DP1,* but perhaps they are produced at the wrong time and/or in inappropriate quantities. The unstable behavior of the *e2f1–1* strain in passing Commitment suggests that it is on the “borderline” for production of such a factor, but once made, it can modulate proper cell division later on in the cell cycle. It is important to note that our assay for Commitment is based operationally on future cell division behavior and is not based on a real-time molecular assay such as presence/absence of a protein modification or production/localization of a protein or RNA. Understanding the molecular details of Commitment and the regulation of cell division number will be critical for truly understanding how the RB pathway mediates cell size control.

The cell size phenotypes we saw for RB pathway mutants in *Chlamydomonas* are similar to the cell size phenotypes of animal and plant cells that have perturbations in the RB pathway [18–21,56,57], but it might be argued that they arise for different reasons—altered size checkpoint control in *Chlamydomonas* versus altered cell cycle kinetics in other organisms. However, the two explanations are not mutually exclusive: Size checkpoint defects in a canonical cell cycle would be expected to alter the overall cell cycle phasing. However, in developing systems, the RB pathway is integrated with developmental control mechanisms at a number of levels, including responses to extracellular signaling pathways, regulation of growth rates, and apoptosis [16,17,23,58,59]. The cell size and developmental phenotypes of an RB mutation in *Dictyostelium*, a primitive slime mold that interconverts between a unicellular and multicellular form, suggest that this integration may be a critical part of the transition to multicellularity [60]. These additional developmental roles do not preclude the participation of the RB pathway in a size checkpoint, but they make the identification of its function as a size regulator more complicated. In animals there has been compelling evidence for a G1-S size checkpoint [3,61] although it may not operate in all cell types [7]. It will be interesting to determine whether the G1 size control that has been observed in animal cells operates through the RB pathway. It is also intriguing that in budding yeast, which has lost the RB pathway, a highly similar genetic mechanism has evolved to control progression through Start, a G1-S size checkpoint [62,63]. In summary, the role we have identified for the RB pathway in size regulation may represent a basal function that evolved in unicellular organisms, to which was added additional regulatory inputs in multicellular species.

### Transcriptional Regulation by the RB Pathway in *Chlamydomonas*

While the genetic architecture of the RB pathway has been conserved in *Chlamydomonas,* its influence on cell cycle transcription has not. The regulation of predicted targets of E2F was not altered significantly in RB pathway mutants, and inhibition of transcription did not influence cell division number. These findings were surprising because many of the transcriptional targets of E2F are conserved between plants and animals [15,41–43]. However, E2F may be bound at many noncanonical sites [64], so predicting targets based on E2F consensus sites may be an inadequate method for identifying E2F-regulated genes. The transcriptional output of the RB pathway usually correlates with cell cycle progression [14,16,17,49,65], but it should be noted that any increase or decrease in the rate of cell cycle progression mediated by changes in RB-E2F activity will indirectly influence levels of cell cycle regulated messages, whether or not they are direct transcriptional targets of E2F. Thus, in asynchronous culture systems it is easy to overestimate the direct influence of the RB pathway on cell cycle transcription. Our ability to synchronize mutant cells allowed us to overcome this problem by examining periodic transcription within a single cell cycle.

It is possible that our experiments were unable to detect a role for E2F1-DP1–mediated transcription in *Chlamydomonas*. Given that RB pathway mutants have defects at Commitment and during S/M, it is possible that the key transcriptional response is for target genes that are activated early in the cell cycle and that these defects carry over into S/M. Another possibility is that the RB pathway does not control the magnitude of cell cycle transcription but its relative timing. In this model, the duration of the transcriptional burst during S/M but not its magnitude would determine cell division number. In other words, the RB pathway would control the window of time during which a cell could initiate S phase and mitosis but would not directly control the induction of S/M-regulated transcripts. In *mat3* mutants, the window would be abnormally lengthened, and in *dp1* mutants, it would be shortened. In any case, our findings do show that the magnitude of S/M transcription does not predict cell division behavior and is not controlled by the RB pathway. This conclusion also implies the existence of an alternative cell cycle transcription mechanism that can induce and repress expression of S/M genes in *Chlamydomonas* independently of the RB pathway.

Despite lacking obvious transcriptional phenotypes, mutants in the RB pathway have very clear cell cycle phenotypes. This discrepancy has prompted us to consider alternative possibilities for how the RB pathway might control the cell cycle. It is conventionally believed that the transcriptional induction of cell cycle genes by E2F-DP complexes has a primary causative role in promoting cell cycle progression. However, the definitive identification of rate-limiting E2F targets is not easy, with the *Drosophila* cyclin E gene being one of few examples [66,67]. Until recently, mammalian cyclin E was also thought to be such a target, but results showing that cyclin E and its binding partner, CDK2, are not essential for cell cycle progression have demonstrated that this is not the case [68–70]. Moreover, there is evidence from *Drosophila* that cell cycle progression and transcription of E2F targets do not always correlate [46,47]. It is, therefore, possible that the primary role of the MAT3/RB pathway in regulating cell cycle progression may not be related to transcription. In this scenario, the RB pathway would activate S phase through some other mechanism, and while we have not identified such a mechanism, there is intriguing evidence suggesting nontranscriptional roles for RB in other organisms. For example, in *Drosophila,* RB complexes have been proposed to regulate chorion gene amplification through direct interaction with origin recognition complexes in a manner that is insensitive to transcriptional inhibition [53,71]. RB has also been found to influence DNA replication in *Xenopus* cell-free extracts by interaction with the replication initiation protein MCM7 [72]. In mammalian cells, RB has been localized to sites of replication during early S phase [73], although this finding has been disputed [74,75]. While evidence exists for nontranscriptional cell cycle regulation by the RB pathway, the relative importance of such regulation has been difficult to assess. Our results suggest that *Chlamydomonas* is an advantageous model in which to address this question.

## Materials and Methods

### Strains and *mat3* suppressor isolation.

Wild-type strain 21gr and *mat3–4* mutants have been described [25]. *mat3–4* suppressors were generated as insertion mutants using the vector pSI103 [76] linearized with NotI and transformed using the glass bead method [77] with selection on 12 μg/ml paromomycin (Sigma, St. Louis, Missouri, United States) on TAP plates [78]. Approximately 20,000 transformed colonies were picked and assayed for growth and color. Those that were faster growing and/or darker green than *mat3–4* were retested for cell size and analyzed further. All candidate mutants were crossed to wild-type strain 6145c, and progeny were tested for linkage of the suppressor phenotype to the pSI103 insertion. Additional details of the insertional screen will be described elsewhere.

Vegetative diploids for dominance testing were constructed as follows: *mt^+^ e2f1–1 mat3–4* was crossed to *mt^−^ mat3–5* (an *mat3* null allele marked by paromomycin resistance) to obtain *mt^−^ e2f1–1 mat3–5* recombinants whose genotypes were confirmed using PCR-based markers. *mt^−^ e2f1–1 mat3–5* or *mt^−^ E2F1 mat3–5* strains (both paromomycin resistant) were mated to *mt^+^ E2F1 mat3–4* (marked by emetine resistance), and vegetative diploids ( *mt^+^/mt^−^ e2f1–1/E2F1 mat3–4*/*mat3–5* or *mt^+^/mt^−^ E2F1/E2F1 mat3–4/mat3–5*) were selected on TAP plates containing 15 μg/ml paromomycin and 60 μg/ml emetine (Sigma) [79,80]. The presence of both mating locus alleles and mutant or wild-type alleles of *MAT3* and *E2F1* was confirmed using PCR on selected diploids [81].

### Culture conditions and synchronization.

Chlamydomonas reinhardtii cells were grown in 500-ml Erlenmeyer flasks in 300 ml of inorganic high-salt medium (HSM) [78] aerated with 0.5% CO_2_ in air with 250 μmol m^−2^ s^−1^ illumination at 24 °C. Otherwise cultures were maintained on TAP plates with 1.5% agar [78].

For dark-shift experiments, cells were grown in continuous light for 2 to 3 d with density maintained at 10^5^ to 10^6^ cells/ml and then transferred into the dark for 16 to 18 h.

Synchrony was induced using the following regimens (Figures 4, 7, and 9): wild-type, *e2f1–1*, *e2f1–1 mat3–4,* and *dp1–1 mat3–4* in 12:12 h light/dark for several weeks (until a high level of synchrony was achieved), followed by transfer to 11:13 h light/dark for several weeks and, through sampling; *dp1–1* in 9:15 h light/dark for several weeks (until a high level of synchrony was achieved) followed by 11:13 h light/dark for several weeks and through sampling. Note that all the strains were well equilibrated in an 11:13 light/dark regimen prior to and during the experiments (Figure 8). Wild-type and *mat3–4* cultures were grown in continuous light and dark-shifted as described above. The cultures were then returned to the light to resume growth (Figure 9A). Wild-type cells were maintained in a 12:12 h light/dark regimen.

For transcriptional inhibition α-amanitin (Axxora LLC, San Diego, California, United States) was added to synchronized wild-type or *dp1–1* cultures at 9 or 8 h in the light period, respectively. For dose-response experiments (Figure 9C and 9D), the cultures were immediately dark-shifted for 18 h, and daughter cell sizes were measured as described below.

### Cell size and cell cycle analysis.

Photomicroscopy was performed using a Deltavision DV3000 system with a ×60 objective and DIC prisms (Applied Precision, Seattle, Washington, United States).

Cell size was determined using samples fixed with 0.2% glutaraldehyde/0.005% Tween 20 and measured using a Coulter Counter (MULTISIZER 3; Beckman-Coulter, Miami, Florida, United States).

Mitotic index and the plate-based Commitment assay were as described previously [25]. Commitment size was measured as described in Figure 3 and Table 1 legends.

For flow cytometry, 10 ml of cells was pelleted in the presence of 0.005% Tween 20 and fixed by resuspending in 10 ml of ethanol/acetic acid (3:1) for 1 h at room temperature. Fixed cells were repelleted and washed once with FACS buffer (0.2 M Tris [pH 7.5], 20 mM EDTA), resuspended in 1 ml of FACS buffer, and stored at 4 °C. Prior to flow cytometry, cells were incubated in FACS buffer at 37 °C with 100 μg/ml RNase A for 2 h, washed once with PBS, and resuspended in 1 ml of PBS. DNA was stained with Sytox Green (Invitrogen, Carlsbad, California, United States) diluted 1:10,000, and cytometry was performed with a FACScan system (Becton-Dickinson, Palo Alto, California, United States).

### Isolation of a genomic *DP1* clone and complementation of *dp1–1.*

Wild-type genomic DNA was double digested with KpnI and BglII (New England Biolabs, Beverly, Massachusetts, United States). A mini- library of approximately 4-kb fragments was generated by ligation into pBluescript SK^−^, and colony lifts were probed with *DP1* sequences (as above). One positive clone (pDP1) was sequenced and used to generate pDP1-ble by inserting the HindIII-digested *ble* marker from pSP124 [82] into the SpeI site in the polylinker of pDP1.

The *DP1-ble* cassette was isolated from pDP1-ble as a KpnI-XhoI fragment and used to transform a *dp1–1 mat3–4* strain using Zeocin selection (5 μg/ml) (Invitrogen). Complemented transformants with *mat3–4*–sized cells were crossed to a wild-type strain (6145c) in order to segregate away the *mat3–4* mutation and to confirm linkage of the Zeocin resistance and complementation phenotypes.

### Isolation and characterization of *e2f1–4.*

*e2f1–4* was isolated during transformation of an *mat3–4 nit1–305* strain [25] with an *NIT1*-containing vector. The inserted *NIT1* DNA in the suppressed transformant was found to be linked to the suppression phenotype and located approximately 10 kb from the *E2F1* locus whose DNA was then sequenced to identify the *e2f1–4* deletion. The deletion removes approximately 1.1 kb of the genomic locus from nucleotides 1124 to 2243 (numbered from the initiator ATG) (corresponding to nucleotides 463 to 1221 of the *E2F1* cDNA) and would generate an in-frame deletion that removes exon 5 and fuses exons 4 and 6. The predicted E2F1–4 protein would be missing amino acids 155 to 407. The library used to isolate *e2f1–4* genomic DNA was made using the λBlueSTAR Vector System (Novagen, Madison, Wisconsin, United States) according to the manufacturer's instructions. Plaque lifts from the library were probed with *E2F1* sequences and positive clones containing *e2f1–4* were isolated and confirmed by sequencing. An approximately 4-kb *e2f1–4* genomic fragment was isolated from a positive phage clone by PspOMI digestion and cloned into the vector pLitmus 38 (New England Biolabs) that had an *aphVIII* cassette encoding paromomycin resistance to generate pE2F1–4. The *aphVIII* cassette was a PvuII-SpeI fragment from pSI103 that had been inserted into the HpaI site of pLitmus 38. pE2F1–4 or control plasmid pSI103 was used to transform an *mat3–4* strain using paromomycin selection (25 μg/ml), and several suppressed clones were chosen from the pE2F1–4 *mat3–4* transformants. Two of the suppressed *mat3–4* transformants were crossed to a wild-type strain (6145c), and the linkage of the cell size phenotype and the paromomycin resistance phenotype was confirmed in the progeny.

### Quantitative RT-PCR.

Approximately 1.5 × 10^7^ cells were pelleted at each time point, flash-frozen in liquid nitrogen, and stored at −70 °C. For *mat3–4,* 4.5 to 5 × 10^7^ cells were collected. RNA was isolated using TRIzol (Invitrogen) according to the manufacturer's instruction. The purified RNA was treated with RNase-free DNase (Qiagen, Valencia, California, United States) followed by RNeasy mini-column purification (Qiagen) according to the manufacturer's instructions.

Total RNA 5 μg was used for cDNA synthesis. cDNA was synthesized at 55 °C for 70 min using ThermoScript RT-PCR (Invitrogen) according to the manufacturer's instructions and using a mixture of dT and random primers (9:1 ratio). Real-time RT-PCR analysis was carried out using a Bio-Rad iCycler iQ (Hercules, California, United States). Each 20-μl RT-PCR contained 0.25 μl of cDNA, 1× ExTaq buffer (Takara, Shiga, Japan), 1× SYBR Green I (Invitrogen), 10 nM fluorescein (Bio-Rad), 0.1% Tween 20, 0.1 mg/ml BSA, 5% DMSO, 200 μM dNTPs, 1 μM primers, and 10 units of Taq polymerase. The following program was used for amplification: 94 °C for 3 min, 40 cycles of 94 °C for 10 s, and 60 °C for 30 s. PCR was performed in triplicate, and the experiments were repeated twice with RNA isolated from independent cultures. A melting curve of each PCR was examined to ensure that no primer-dimers or spurious products were present.

### Southern blot analyses.

Genomic DNA preparation and Southern blotting were carried out as previously described [24]. Probes were derived from PCR-amplified cDNA using primers 5′-CCATGAAGGTGTGCGAAAAGG-3′ and 5′-CTGCACGTCCATCATGTCGTC-3′ for *DP1*, and primers 5′-CTGAAACGTTGAAGGTCCAAA-3′ and 5′-ACCAGGTACACGTCGATGG-3′ for *E2F1*.

## Supporting Information

Figure S1 Southern Blot of Genomic DNA from Different Strains (Indicated above Each Lane) Digested with SmaI and Probed with *DP1* or *E2F1* DNA *dp1–1* contains an insertion in the *DP1* locus. *dp1–3, −4 ,-6, −7, −8* alleles have deletions that remove *DP1* and some flanking sequence. *e2f1–1, −2, −3* contain insertions in a 1.4-kb SmaI fragment depicted in Figure 1B.
(690 KB TIF)

Figure S2 Assessment of *DP1* and *E2F1* Copy Number by Low-Stringency Southern blotting (A) Restriction maps of *DP1* and *E2F1* genomic DNA. The predicted DNA fragments digested with BamHI, NheI, or SmaI are indicated.
(B) Low-stringency Southern blot of wild-type genomic DNA digested with BamHI (B), NheI (N), or SmaI (S) and probed with cDNA fragments corresponding to the conserved N-terminal regions of DP1 or E2F1. Low-stringency blotting was carried out as previously described [24].
(1.3 MB TIF)

Figure S3 Click here for additional data file. Cell Size and Semiquantitative RT-PCR Analysis of *dp1–3*, a Deletion Allele
(A) Size distribution of dark-shifted cultures from indicated the genotypes.
(B) RT-PCR of *MCM2, RNR1, PCN1, POLA4, CYCA1, CYCB1, CDKB1,* and *TUA1* in unsynchronized light-grown cultures of the indicated genotype as described in [24]. α-Tubulin *(TUA1)* serves as the internal control. The percent mitotic state indicates what fraction of cells in the culture were mitotic when the sample was taken for RNA preparation. The mitotic fraction was higher for the *dp1* mutants compared to wild-type and explains the increased abundance of cell cycle messages in the mutant strains compared to the wild-type control.
(837 KB TIF)

Figure S4 Click here for additional data file. Expression Patterns of *CYCA1, CYCB1, CDC6, PCN1, RIR1, MCM2,* and *HFO* in Synchronized Cultures of Indicated Genotype
The samples are from the same cultures as in Figure 7.
(688 KB TIF)

Figure S5 Click here for additional data file. Expression Patterns of *CYCB1, CDC6, PCN1, RIR1, MCM2,* and *HFO* in Partially Synchronized Cultures of Wild-Type and *mat3–4* Strains
The samples used are the same as in Figure 8.
(298 KB TIF)

Table S1 List of Primers for Quantitative RT-PCR (21 KB DOC)

### Accession Numbers

The GenBank (http://www.ncbi.nlm.nih.gov/Genbank) accession numbers for DP1 and E2F1 are DQ417491 and DQ417492.

## Abbreviations

RB: retinoblastoma
RNAi: RNA interference
