# Supplementary material for: Cell Size Checkpoint Control by the Retinoblastoma Tumor Suppressor Pathway
Source: PLoS Genet. 2006 Oct 13;2(10):e167. doi: 10.1371/journal.pgen.0020167 (PMC1599770; doi:10.1371/journal.pgen.0020167)
Supplement: Supplementary file 1 [file pgen.0020167.st001.doc]

|  | **RT-PCR primers 5’ to 3’:** |
| --- | --- |
| 18SrRNA-1 | ATCTGCGAAAGCATTTGCCA |
| 18SrRNA-2 | CGGCATCGTTTATGGTTGAGAC |
| CYCA1-3 | GTCGGCGATTAACTACTCCCAG |
| CYCA1-4 | CCGTTGCTAGATACTCCCTTGC |
| CYCB1-3 | GGTGCACCTGAAGTTCAAGCTG |
| CYCB1-4 | AATCTCCTCGTACTTGGACGCG |
| CDKB1-3 | GACAACGCTGCGTGAGATTTC |
| CDKB1-4 | ACCAGGTAAAGGCATGGCTTG |
| PCN1-3 | GCCTTCCACGTCTCACAATCAT |
| PCN1-4 | GCTGACATCGAAATTGCCCTC |
| POLA4-3 | AGGCACAGATGGACCGAGAAT |
| POLA4-4 | TTCCGTAGGACAGCCACTTCAC |
| HFO-1 | AGCGTATCAGCGGCCTCATCTA |
| HFO-2 | GGATGACGTTCTCCAGGAAGGT |
| RIR1-3 | TTGACAAGATTACAGCGCGC |
| RIR1-4 | GACACCCTTGTAGACACCACCA |
| MCM2-3 | TTCTTTGATGCGTTCAAGGC |
| MCM2-4 | GAGGCAGTTGAGGTGGTAGTTG |
| CDC6-1 | CATGGTCTTCACCGCATACAG |
| CDC6-2 | ACGGACTTTGCCACCATGT |
